# Abnormal Microstructural Development of the Cerebral Cortex in Neonates With Congenital Heart Disease Is Associated With Impaired Cerebral Oxygen Delivery

**DOI:** 10.1161/JAHA.118.009893

**Published:** 2019-03-01

**Authors:** Christopher J. Kelly, Daan Christiaens, Dafnis Batalle, Antonios Makropoulos, Lucilio Cordero‐Grande, Johannes K. Steinweg, Jonathan O'Muircheartaigh, Hammad Khan, Geraint Lee, Suresh Victor, Daniel C. Alexander, Hui Zhang, John Simpson, Joseph V. Hajnal, A. David Edwards, Mary A. Rutherford, Serena J. Counsell

**Affiliations:** ^1^ Centre for the Developing Brain School of Biomedical Engineering and Imaging Sciences King's College London St Thomas’ Hospital London United Kingdom; ^2^ Biomedical Image Analysis Group Department of Computing Imperial College London London United Kingdom; ^3^ Department of Forensic and Neurodevelopmental Sciences King's College London Institute of Psychiatry, Psychology and Neuroscience London United Kingdom; ^4^ Department of Neuroimaging King's College London Institute of Psychiatry, Psychology and Neuroscience London United Kingdom; ^5^ MRC Centre for Neurodevelopmental Disorders King's College London London United Kingdom; ^6^ Neonatal Intensive Care Unit St Thomas’ Hospital London United Kingdom; ^7^ Department of Computer Science and Centre for Medical Image Computing University College London London United Kingdom; ^8^ Paediatric Cardiology Department Evelina London Children's Hospital St Thomas’ Hospital London United Kingdom

**Keywords:** brain imaging, cerebral blood flow, congenital heart disease, development, magnetic resonance imaging, Congenital Heart Disease, Magnetic Resonance Imaging (MRI), Imaging, Pediatrics, Cognitive Impairment

## Abstract

**Background:**

Abnormal macrostructural development of the cerebral cortex has been associated with hypoxia in infants with congenital heart disease (CHD). Animal studies have suggested that hypoxia results in cortical dysmaturation at the cellular level. New magnetic resonance imaging techniques offer the potential to investigate the relationship between cerebral oxygen delivery and cortical microstructural development in newborn infants with CHD.

**Methods and Results:**

We measured cortical macrostructural and microstructural properties in 48 newborn infants with serious or critical CHD and 48 age‐matched healthy controls. Cortical volume and gyrification index were calculated from high‐resolution structural magnetic resonance imaging. Neurite density and orientation dispersion indices were modeled using high‐angular‐resolution diffusion magnetic resonance imaging. Cerebral oxygen delivery was estimated in infants with CHD using phase contrast magnetic resonance imaging and preductal pulse oximetry. We used gray matter–based spatial statistics to examine voxel‐wise group differences in cortical microstructure. Microstructural development of the cortex was abnormal in 48 infants with CHD, with regions of increased fractional anisotropy and reduced orientation dispersion index compared with 48 healthy controls, correcting for gestational age at birth and scan (family‐wise error corrected for multiple comparisons at *P*<0.05). Regions of reduced cortical orientation dispersion index in infants with CHD were related to impaired cerebral oxygen delivery (*R* ^2^=0.637; n=39). Cortical orientation dispersion index was associated with the gyrification index (*R* ^2^=0.589; *P*<0.001; n=48).

**Conclusions:**

This study suggests that the primary component of cerebral cortex dysmaturation in CHD is impaired dendritic arborization, which may underlie abnormal macrostructural findings reported in this population, and that the degree of impairment is related to reduced cerebral oxygen delivery.

**Clinical Perspective**

**What Is New?**

- Newborn infants with serious or critical congenital heart disease demonstrate abnormal development of the cerebral cortex at the microstructural level, assessed using diffusion magnetic resonance imaging.
- This study suggests that the primary component of cerebral cortex dysmaturation in congenital heart disease is impaired dendritic arborization.
- Impairment of cortical microstructural development was associated with reduced cerebral oxygen delivery measured in the newborn period.

**What Are the Clinical Implications?**

- Identification of abnormal development of the cerebral cortex in this population provides insight into the mechanisms that may underlie poorer neurodevelopmental outcomes in congenital heart disease.
- The relationship between impaired cerebral oxygen delivery and abnormal cortical microstructure corroborates recent animal models investigating the role of oxygen tension in cortical development.
- Methods to quantitatively assess impairment of cortical development may enable more rapid assessment and iteration of novel interventions to improve the trajectory of brain development in congenital heart disease during pregnancy.

## Introduction

Congenital heart disease (CHD) is the most common congenital abnormality, affecting almost 1% of newborns.^1^ Despite improvements in antenatal diagnosis, cardiac surgery, and perioperative care, infants with CHD often experience neurological impairment across a range of developmental domains, both in early childhood and later in adult life,^2^, ^3^ making improvement of neurodevelopmental outcomes a major remaining challenge in the management of CHD. While early research focused on suspected surgical and perioperative factors, it now appears that a more complex set of biological factors may be responsible.

The detrimental effect of CHD on early brain development can be observed via a faltering trajectory of brain growth in the third trimester of pregnancy^4^, ^5^, ^6^, ^7^ and a higher incidence of acquired brain lesions in newborn infants before cardiac surgery.^8^ The developmental morphology of the cortex has become of increasing interest in CHD, with an “immature cortical mantle” first observed in autopsies of infants with hypoplastic left heart syndrome.^9^ Reduced cortical folding has since been quantified in vivo using magnetic resonance imaging in both fetal^6^ and presurgical neonatal populations.^10^, ^11^, ^12^ Hypothesized contributory factors include reduced fetal cerebral oxygen delivery^5^ and cerebral metabolic substrate,^13^ although precise cellular mechanisms remain unclear.

Linking physiological changes in CHD to brain development is assisted by 4 recent findings. First, oxygen tension has been shown to regulate development of human cortical radial glial cells, with hypoxia exerting negative effects on gliogenesis by reducing the number of preoligodendrocytes while increasing the number of reactive astrocytes.^14^ Second, hypoxia has been shown to reduce proliferation and neurogenesis in the subventricular zone of the piglet brain, accompanied by reduced cortical growth, with preliminary similarities found in the subventricular zone cytoarchitecture in human fetal autopsy specimens.^15^ Third, ascending aorta oxygen saturations have been found to be 10% lower in human fetuses with mixed CHD compared with healthy controls, with saturation measurements that correlated with fetal brain size.^5^ Finally, microstructural maturation of the cortex, measured using both histology and diffusion anisotropy, has been demonstrated to occur in parallel with macrostructural development.^16^ Taken together, these studies raise the hypothesis that macrostructural changes observed in CHD are the result of altered cortical microstructural development, which is in turn hindered by suboptimal oxygen tension during fetal life in CHD. Diffusion magnetic resonance imaging, with newer multicompartment models such as neurite orientation dispersion and density imaging (NODDI), provides measures that enable this hypothesis to be tested. Diffusion tensor imaging metrics such as fractional anisotropy (FA) are nonspecific and reflect many underlying parameters of brain tissue including neuronal density, fiber orientation dispersion, degree of myelination, free‐water content, and axonal diameter.^17^ The NODDI model aims to disentangle these different factors by separating the influence of neurite density and orientation dispersion from each other, and from partial volume with cerebrospinal fluid, to provide distinct indices: orientation dispersion index (ODI), which captures the degree of dispersion of axonal fiber orientations (eg, through fanning, bending, crossing) or dendrite orientations, and neurite density index (NDI), represented by the intracellular volume fraction.^18^

In this study, we aimed to use high‐angular‐resolution diffusion imaging and NODDI to test the hypothesis that reduced cerebral oxygen delivery in CHD is associated with impaired cortical microstructural development. We predicted that infants with CHD would exhibit higher cortical FA and lower ODI when compared with a group of healthy matched controls, and that infants with the lowest cerebral oxygen delivery would exhibit the most severe impairment of cortical microstructural development.

## Methods

The project was approved by the National Research Ethics Service West London committee (CHD: 07/H0707/105; Controls: 14/LO/1169) and informed written parental consent was obtained before imaging. All methods and experiments were performed in accordance with relevant guidelines and regulations. The data, analytic methods, and study materials will be available to other researchers for purposes of reproducing the results or replicating the procedure on reasonable request.

### Participants

A prospective cohort of 54 infants with serious or critical CHD^19^ expected to require surgery within 1 year was recruited after birth from the neonatal intensive care unit at St Thomas’ Hospital, London. Infants were excluded if they appeared phenotypically abnormal other than the congenital heart defect, had a suspected or confirmed chromosomal abnormality, any previous neonatal surgery, or who had a suspected congenital infection. Six infants were excluded from the analysis: 2 infants with suspected coarctation were later assessed to have a normal circulation following postnatal ductus arteriosus closure; 2 infants were found to have focal arterial ischemic stroke on magnetic resonance imaging involving the cortex (both left middle cerebral artery stroke); 1 infant had uncertain gestation attributable to unknown date of last menstrual period and lack of ultrasound dating scan; 1 infant had incomplete diffusion data attributable to waking during the scan.

We therefore studied 48 infants with CHD, born at a median gestational age (GA) of 38.8 weeks (interquartile range, 38.0–39.1). A control group of 48 healthy infants was retrospectively matched to the CHD group by GA at birth and scan, born at a median GA of 38.5 weeks (38.1–38.9). Healthy infants were recruited contemporaneously from the postnatal ward at St Thomas’ Hospital as part of the Developing Human Connectome Project.^20^ The median GA at scan was 39.1 weeks (interquartile range, 38.6–39.7) for both the CHD group and control group. Twenty‐six infants with CHD were on a prostaglandin infusion to maintain ductal patency at the time of scan (54%).

### Magnetic Resonance Imaging

T1‐weighted (T1w), T2‐weighted (T2w), diffusion‐weighted imaging (DWI), and phase contrast angiography magnetic resonance imaging was performed on a Philips Achieva 3 Tesla system (Best, The Netherlands) with a 32‐channel neonatal head coil and neonatal positioning device,^20^ situated in the neonatal intensive care unit at St Thomas’ Hospital, London. All examinations were supervised by a pediatrician experienced in magnetic resonance imaging procedures. All infants were scanned in natural sleep without sedation. Pulse oximetry, respiratory rate, temperature, and electrocardiography were monitored throughout. Ear protection comprised earplugs molded from a silicone‐based putty (President Putty, Coltene Whaledent, Mahwah, NJ) placed in the external auditory meatus, neonatal earmuffs (MiniMuffs, Natus Medical Inc, San Carlos, CA) and an acoustic hood positioned over the infant. All sequences included a 5‐second initial slow ramp‐up in acoustic noise to avoid eliciting a startle response.

T2w images were acquired using a multislice turbo spin echo sequence, acquired in 2 stacks of 2‐dimensional slices (in sagittal and axial planes), using parameters: repetition time: 12 seconds; echo time: 156 milliseconds, flip angle: 90°, slice thickness: 1.6 mm acquired with an overlap of 0.8 mm; in‐plane resolution: 0.8×0.8 mm, scan time: 3:12 minutes per stack. The T1w volumetric magnetization prepared rapid acquisition gradient echo acquisition parameters were as follows: repetition time: 11 milliseconds, echo time: 4.6 milliseconds, TI: 714 milliseconds, flip angle: 9°, acquired voxel size: 0.8×0.8×0.8 mm, field of view: 145×145×108 mm, sensitivity encoding factor: 1.2, scan time: 4:35 minutes. DWI with 300 directions was acquired using parameters: repetition time: 3.8 seconds, echo time: 90 milliseconds, multiband: 4; sensitivity encoding E: 1.2; resolution: 1.5×1.5×3 mm with 1.5 mm slice overlap, diffusion gradient encoding: b=0 s/mm (n=20), b=400 s/mm (n=64), b=1000 s/mm (n=88), b=2600 s/mm (n=128) with interleaved phase encoding.^21^ Quantitative flow imaging was performed using velocity‐sensitized phase contrast imaging, with a single‐slice T1w fast field echo sequence. Scan parameters were: field of view: 100×100 mm, acquisition resolution: 0.6×0.6×4.0 mm, repetition time: 6.4 milliseconds, echo time: 4.3 milliseconds, flip angle: 10°, 20 repetitions, maximal encoding velocity: 140 cm/s, scan time: 71 seconds.^22^

### Structural and Diffusion‐Weighted Image Reconstruction

T2w images were reconstructed using a dedicated neonatal motion correction algorithm. Retrospective motion‐corrected reconstruction^23^, ^24^ and integration of the information from both acquired orientations^25^ were used to obtain 0.8 mm isotropic T2w volumes with significantly reduced motion artifacts. Diffusion images were reconstructed following the scan using a dedicated multiband reconstruction method described previously.^21^

### Structural Image Processing

Motion‐corrected T2w images were segmented into tissue type using an automated, neonatal‐specific pipeline,^26^, ^27^, ^28^ which was optimized for our acquisition parameters. Each tissue segmentation was manually inspected for accuracy using ITK‐SNAP software,^29^ and minor corrections performed if necessary. The gyrification index (GI) was calculated as described previously.^12^

### Diffusion‐Weighted Image Processing

High‐angular‐resolution diffusion‐weighted imaging data were reconstructed using a slice‐to‐volume motion correction technique that uses a bespoke spherical harmonics and radial decomposition of multishell diffusion data, together with outlier rejection, distortion, and slice profile correction.^30^, ^31^ Data were first processed with image denoising^32^ and Gibbs ringing suppression.^33^ A field map was estimated from b=0 images using Topup from the FMRIB Software Library (FSL).^34^ Reconstruction was run for 10 iterations with Laplacian regularization, using a spherical harmonics and radial decomposition decomposition of rank=89 (allowing for spherical harmonics order ℓ_max_=0, 4, 6, 8 for respective shells), with registration operating at a reduced rank=15.

Nonbrain tissue was removed using FSL BET (Brain Extraction Tool).^35^ Diffusion tensor imaging metrics FA and MD were calculated from b=0 and b=1000 DWI data using MRtrix3.^36^ NODDI parameter maps were estimated using NODDI toolbox version 0.9.^18^ We performed a Bayesian information criterion comparison to compare the quality of the NODDI model fit for different intrinsic diffusivity values, which was found to be optimal at 2.0×10^−3^ mm^2^ s^−1^. This is consistent with previous NODDI studies in neonates,^37^, ^38^, ^39^ with the higher value compared with adults (usually 1.7×10^−3^ mm^2^ s^−1^) likely reflecting the higher water content of the neonatal brain.

### Group Template Generation and Image Registration

A multivariate group template was generated from both T1w and T2w images, using symmetric diffeomorphic normalization for multivariate neuroanatomy and a cross‐correlation similarity metric.^40^ Each subject's diffusion data were rigidly registered to each subject's T2w image using the MD map.^41^ Diffusion tensor imaging and NODDI maps were then transformed into template space in a single step using concatenated linear and diffeomorphic transformations. Tissue segmentations from T2w images were also transformed into template space using nearest neighbor interpolation.

### Cortical Analysis of Microstructure

We used an approach for aligning cortical data from multiple subjects into a common space to provide voxel‐wise spatial characterization of FA, MD, NDI, and ODI, as previously described.^42^, ^43^ A mean cortical map was produced by merging cortical gray matter segmentations that had been transformed previously into template space. This was then skeletonized to retain only a core of highly probable cortical voxels, represented as a thin curved surface at the center of the cortex. FA, MD, NDI, and ODI measurements from each individual were projected onto the cortical skeleton by searching in a direction perpendicular to the cortical skeleton to identify voxels with the highest probability of being cortical.

### Calculation of Cerebral Oxygen Delivery

For infants with CHD, we calculated their cerebral blood flow using a previously described method.^12^ Phase contrast angiography was acquired in a plane perpendicular to both internal carotids and basilar arteries at the level of the sphenoid bone.^22^ Hemoglobin measurements were performed as part of routine clinical care, at a median of 3 days (interquartile range, 0–5) before the scan. SaO_2_ was measured at the time of scan using a Masimo Radical‐7 monitor (Masimo Corp, Irvine, CA) applied to the right hand.

Cerebral oxygen delivery (CDO_2_) was calculated using the following formula:^44^

$${\text{CDO}}_{2}\;\left(\text{mL}\;O_{2}/\text{min}\right)={\text{SaO}}_{2}\times \left[\text{Hb}\right]\;\left(\text{g/dL}\right)\times 1.36\times \left[\text{CBF}\right]\;\left(\text{mL/min}\right)$$

where 1.36 is the amount of oxygen bound per gram of hemoglobin at 1 atmosphere (Hüfner's constant).^45^

### Statistical Analysis

The control group was retrospectively matched to the CHD group by GA at birth and at scan using an R implementation^46^ of the daisy algorithm^47^ to minimize group differences in age. Statistical tests to perform group comparisons were undertaken using standard unconditional analyses. To investigate the relationship between DWI metrics in the cortical gray matter and clinical factors, cross‐subject voxel‐wise statistical analysis was performed using FSL Randomise v2.9,^43^ using 10 000 iterations of a random permutation method that employed threshold‐free cluster enhancement^48^ based on a general linear model (GLM) design matrix. GA at birth and scan were included as covariates in each model when comparing group differences in brain volume and diffusion measures between infants with CHD and healthy controls. The effect of CDO_2_ on cortical diffusion metrics was assessed using a GLM that selected only infants with CHD who had a successful CDO_2_ measurement (n=39). All analyses were subject to family‐wise error correction for multiple comparisons, and thresholding for all analyses was at *P*<0.05. Linear regression was used to investigate the association between GI and diffusion metrics. To assess the relationship of GI and ODI independently of advancing brain maturity, GA at scan was included as a variable in the multiple linear regression model.

Categorical clinical variables were compared using Fisher's exact tests. For continuous clinical variables, we determined medians and interquartile ranges, and compared groups using the Mann–Whitney *U* test. All analyses of clinical variables were performed using SPSS V24 (IBM, New York).

## Results

The analysis included 96 newborn infants: 48 infants with confirmed serious or critical CHD scanned before surgery without evidence of arterial ischemic stroke, and 48 age‐matched healthy infants. Clinical characteristics of both groups are shown in Table 1. The 2 most common diagnoses were transposition of the great arteries (n=21; 44%), and coarctation of the aorta (n=9, 19%; 7/9 on a prostaglandin infusion at time of scan; 2/9 being monitored off prostaglandin with a patent duct; all required surgical repair in the neonatal period). A summary of preductal saturations and cerebral blood flow and oxygen delivery measurements is displayed in Table 2. There were no significant differences in GA at birth, GA at scan, and sex between groups. T1w, T2w, and DWI were acquired in all infants. An antenatal diagnosis had been made in 47 infants (98%), and no infants experienced cardiorespiratory collapse before the scan. Phase contrast angiography was acquired with acceptable quality in 81% of infants with CHD (n=39). Punctate white matter lesions were present in 15 infants, all of whom had CHD (31%; n=15/48; Table S1).

**Table 1:** Clinical Characteristics of the CHD and Control Cohorts

Variable	Control Newborns (n=48)	Newborns With CHD (n=48)	*P* Value
Gestational age at birth, wks	38.8 (38.0–39.1)	38.5 (38.1–38.9)	0.543
Gestational age at scan, wks	39.1 (38.6–39.7)	39.1 (38.6–39.7)	0.595
Male sex, n (%)	26 (54)	27 (56)	1.000
Birth weight, kg	3.17 (2.83–3.41)	3.10 (2.81–3.47)	0.809
Birth head circumference, cm	34.0 (33.0–35.0)	34.0 (33.0–35.0)	0.500
Heart lesion, n (%)			
TGA	···	21 (44)	
TGA requiring septostomy (% TGA)	···	9 (43)	
Coarctation of the aorta	···	9 (19)	
Tetralogy of Fallot	···	7 (15)	
Severe pulmonary stenosis	···	3 (6)	
Hypoplastic left heart syndrome	···	3 (6)	
Pulmonary atresia	···	3 (6)	
Truncus arteriosus	···	1 (2)	
Tricuspid atresia		1 (2)	

Values presented as median (interquartile range) unless otherwise stated. *P*‐values calculated using Mann–Whitney *U* test for continuous data and Fisher's exact test for categorical variables (sex). Septostomy in TGA was performed before imaging in all cases. CHD indicates congenital heart disease; TGA, transposition of the great arteries.

**Table 2:** Preductal SpO_2_ (Measured Using the Right Hand) on Initial Admission to the Neonatal Intensive Care Unit After Birth and at the Time of Scan, and Cerebral Blood Flow and Cerebral Oxygen Delivery at Time of Scan

Diagnosis	n	Admission Preductal SpO_2_	Preductal SpO_2_ at Scan	On Prostaglandin Infusion at Time of Scan, n (%)	Cerebral Blood Flow Measurement (n)	Cerebral Blood Flow (mL/min)	Cerebral Oxygen Delivery (mL O_2_/min)
TGA	21	85 (75–89)	87 (84–94)	11 (52)	18	77.6 (71.0–109.8)	1615 (1346–1928)
Coarctation of aorta	9	97 (96–98)	99 (98–99)	7 (78)	7	101.1 (89.0–116.3)	2200 (1627–2322)
Tetralogy of Fallot	7	93 (92–97)	94 (90–98)	2 (29)	6	93.8 (84.6–109.3)	2345 (2006–2565)
Hypoplastic left heart syndrome	3	91 (88–98)	95 (90–98)	3 (100)	1	65.4	1285
Pulmonary atresia	3	90 (90–91)	86 (75–91)	3 (100)	3	109.4 (100.3–118.8)	1757 (1471–2680)
Severe pulmonary stenosis	3	94 (91–97)	86 (84–88)	0 (0)	2	90.6 (82.3–98.9)	1519 (1413–1625)
Tricuspid atresia	1	94	96	0 (0)	1	57.0	1168
Truncus arteriosus	1	93	100	0 (0)	1	58.8	1120
Total	48	91 (85.5–96)	98 (92–98)	26 (54)	39	89.0 (71.1–109.4)	1657 (1418–2226)

All values unless otherwise stated are median (interquartile range). TGA indicates transposition of the great arteries.

### Cortical ODI Is Reduced in Infants With CHD

**Figure 1:**
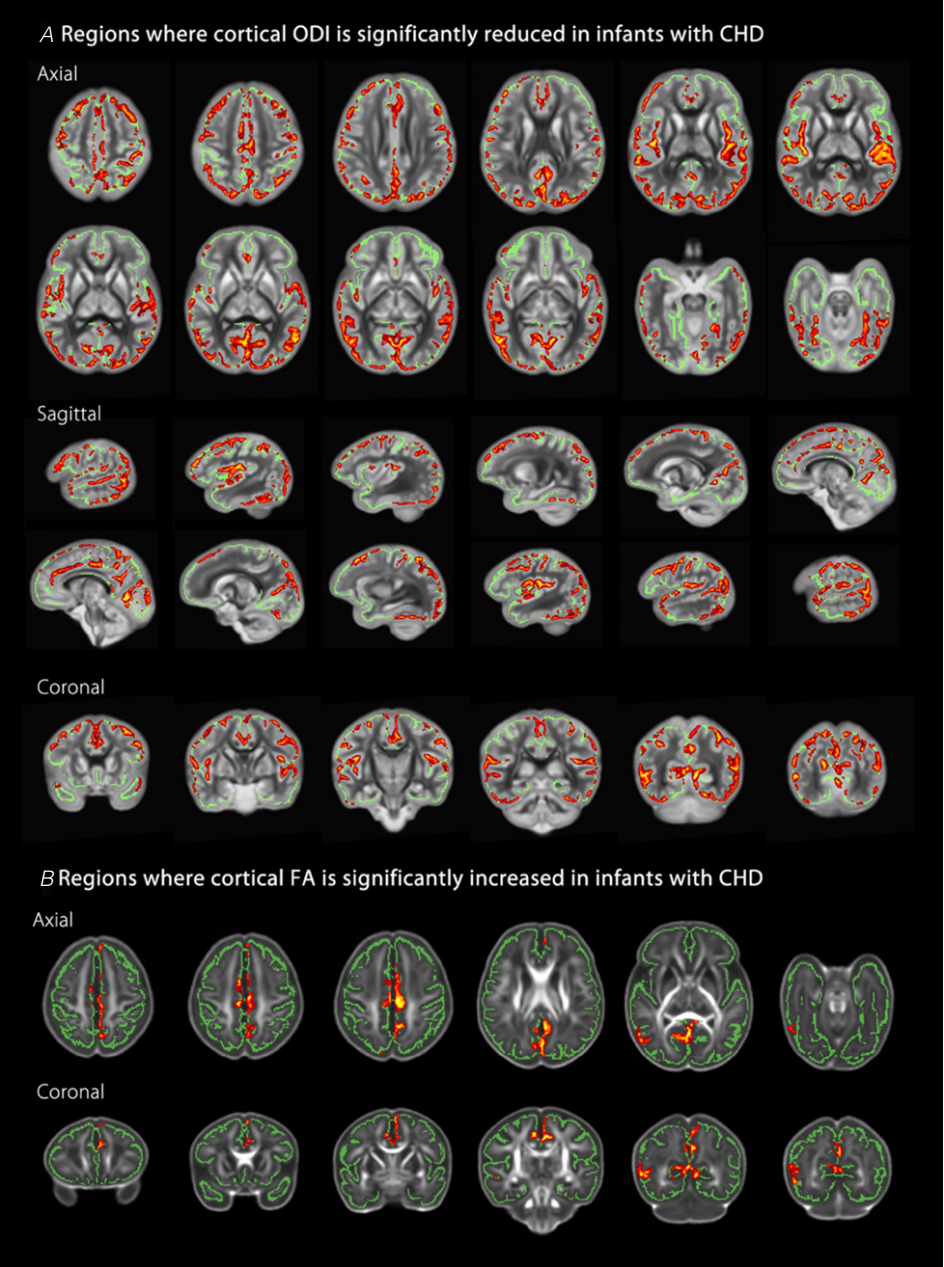
Infants with congenital heart disease (CHD) (n=48) exhibit impaired microstructural development compared with healthy age‐matched controls (n=48). **A**, Regions where orientation dispersion index (ODI) is significantly reduced in infants with CHD, overlaid on the mean ODI template. **B**, Regions where cortical fractional anisotropy (FA) is significantly increased in infants with CHD, overlaid on the mean FA template. Red‐Yellow indicates *P*<0.05 after family‐wise error correction for multiple comparisons following threshold‐free cluster enhancement. Results are shown overlaid on the mean cortical skeleton (green). Cross‐subject voxel‐wise statistical analysis performed using FSL Randomise v2.943, with a general linear model (GLM) used to assess group differences between diffusion measures of infants with CHD and healthy controls. Both analyses included gestational age at birth and at scan as covariates. Number of permutations was 10 000. Left–right orientation is according to radiological convention.

Infants with CHD demonstrated widespread changes in cortical ODI, with the most significant reductions observed posteriorly in the posterior parietal cortex, insula cortex, cingulate cortex, primary motor cortex, supplementary motor area, and occipital regions (Figure 1A, GLM including GA at birth and scan as covariates). Mean cortical ODI values from regions of difference are illustrated in Figure S1A and Table S2. There were no regions where ODI was significantly higher in infants with CHD. There were no differences in NDI between groups. GA at scan demonstrated a widespread positive association with cortical ODI (GLM including GA at birth and scan as covariates). Subgroup analysis of those operated in the first 30 days after birth (n=37) demonstrated a similar distribution of impaired cortical ODI in those with CHD (Figure S2).

### Cortical FA Is Higher in Infants With CHD

Cortical FA was higher in infants with CHD, with effects seen in predominantly midline cortical structures (Figure 1B, GLM including GA at birth and scan as covariates). Mean cortical FA values from regions of difference are illustrated in Figure S1B and Table S2. There were no regions where FA was significantly lower in infants with CHD. There were no differences in mean diffusivity between groups. Cortical FA demonstrated a widespread negative association with GA at scan (GLM including GA at birth and scan as covariates).

### Reduced Cerebral Oxygen Delivery Is Associated With Impaired Cortical Dispersion

**Figure 2:**
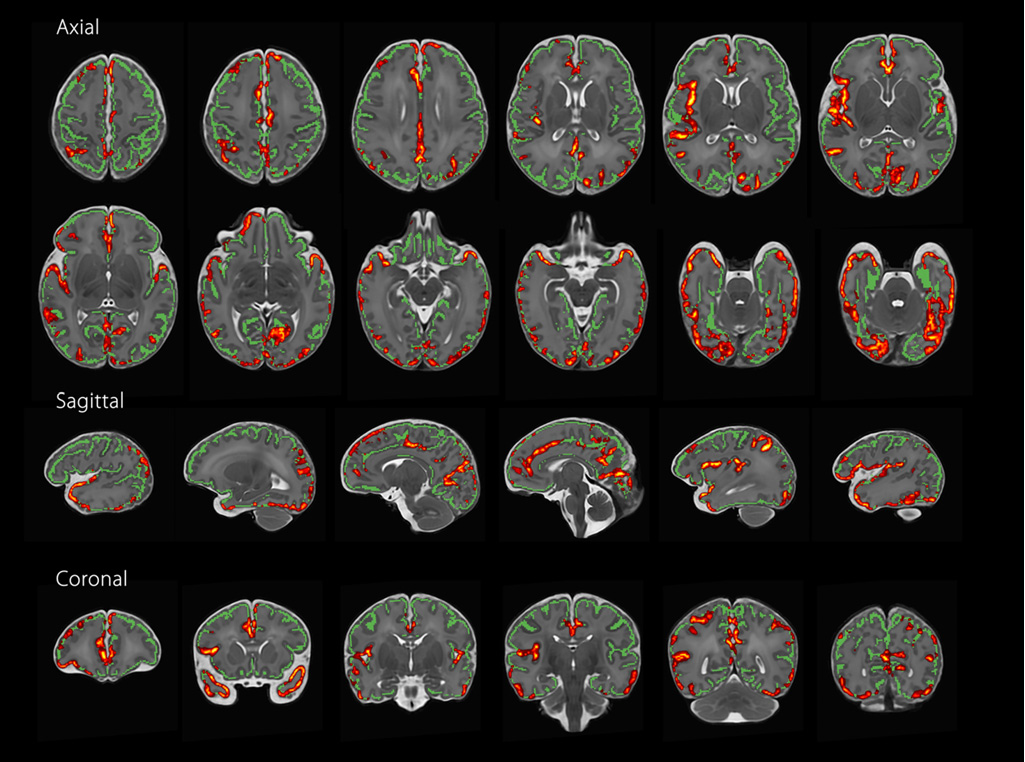
Regions where cerebral oxygen delivery is positively associated with cortical orientation dispersion index in infants with congenital heart disease (n=39). Red‐Yellow indicates *P*<0.05 after family‐wise error correction for multiple comparisons following threshold‐free cluster enhancement. Results are shown overlaid on the group T2‐weighted template and the mean cortical skeleton (green). Cross‐subject voxel‐wise statistical analysis performed using FSL Randomise v2.9.^43^ Number of permutations was 10 000. Left–right orientation is according to radiological convention.

**Figure 3:**
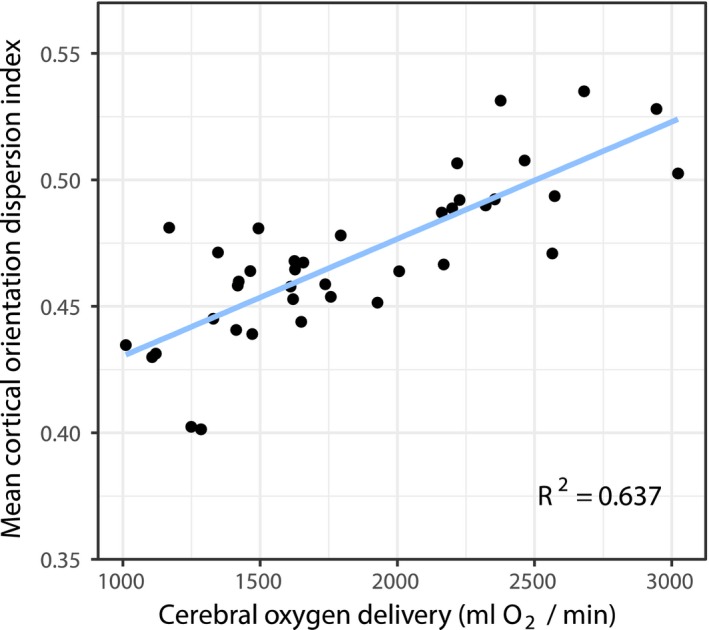
Visualization of the linear relationship between cerebral oxygen delivery (CDO_2_) and cortical orientation dispersion index (ODI) within significant voxels from analysis displayed in Figure 2 (n=39). Mean ODI data were extracted for each subject from significant voxels in the statistic image after family‐wise error correction for multiple comparisons at *P*<0.05.

Cerebral oxygen delivery (CDO_2_) at time of scan was positively associated with cortical ODI across many regions of the cortex (Figure 2, family‐wise error corrected for multiple comparisons, *P*<0.05), with the most significant associations found in the bilateral temporal lobes, occipital lobes, cingulate cortex, and right insula cortex. To demonstrate this linear relationship, mean ODI data were extracted for each subject from significant voxels in the gray matter skeleton and plotted against CDO_2_ at the time of scan (*R* ^2^=0.637, Figure 3). There were no voxels with a negative association between the 2 variables. To assess the relative contribution of each component of CDO_2_, we repeated the analysis substituting CDO_2_ for either cerebral blood flow or preductal arterial saturation at time of scan. Considered alone, neither component demonstrated voxels that reached significance for either a positive or negative relationship with ODI. Repeating these voxel‐wise analyses to investigate associations between CDO_2_ and FA, MD or NDI revealed no significant regions. Summary measures of cerebral blood flow and CDO_2_ are presented in Table 2.

### Relationship Between Cortical Microstructure and Macrostructure in CHD

**Figure 4:**
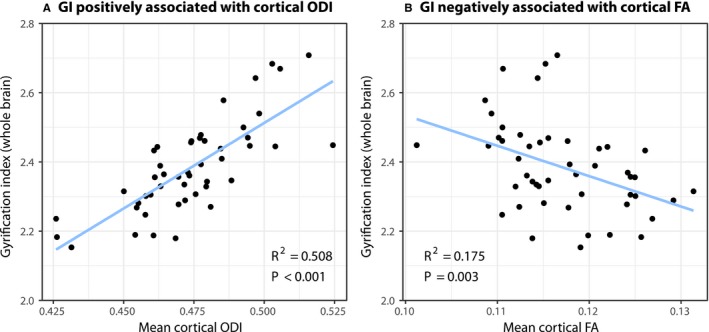
The linear relationship between the gyrification index (GI) and cortical diffusion measures of the orientation dispersion index (ODI) and fractional anisotropy (FA) in newborn infants with congenital heart disease (n=48). GI is (A) positively associated with cortical ODI and (B) negatively associated with cortical FA.

We have previously reported reduced brain volumes and GI in our cohort of infants with CHD.^12^ However, the relationship between cortical microstructure and macrostructure has not been assessed in this group. We found that GI was significantly positively associated with mean cortical ODI (linear regression model; *R* ^2^=0.589; *P*<0.001) and negatively with FA (*R* ^2^=0.175; *P*=0.003) (Figure 4). The linear relationship between GI and cortical ODI persisted following inclusion of GA at scan in the linear regression model (β=0.642; *P*<0.001) but not for cortical FA (β=−0.304; *P*=0.090). Cortical gray matter volume was significantly positively correlated with cortical ODI (*R* ^2^=0.170; *P*=0.004) but not with cortical FA (*R* ^2^=0.060; *P*=0.094). Results are summarized in Table 3. Total brain volume was significantly reduced in those with CHD compared with controls (general linear model including GA at scan as a covariate; 311 mL versus 330 mL; *P*=0.005), and regional brain volumes were significantly smaller in those with CHD across all regions of the brain (GLM including GA at scan as a covariate; Table S2).

**Table 3:** Summary of Linear Regression Analysis of Cortical Microstructural and Macrostructural Measures in Those With Congenital Heart Disease (n=48)

	FA	ODI	Description
Gyrification index	*R* ^2^=0.175, *P*=0.003^a^	*R* ^2^=0.589, *P*<0.001^a^	Linear regression model
Gyrification index	β=−0.304, *P*=0.090	β=0.642, *P*<0.001^a^	Linear regression model including GA at scan
Cortical gray matter volume	*R* ^2^=0.060, *P*=0.094	*R* ^2^=0.170, *P*=0.004^a^	Linear regression model

Model type used is illustrated for each analysis. FA indicates fractional anisotropy; GA, gestational age; ODI, orientation dispersion index.

a A significant result using *P*=0.0083 as threshold following Bonferroni correction.

## Discussion

Long‐term neurodevelopmental impairment is a major remaining challenge for infants with congenital heart disease, yet our understanding of the underlying biological substrate remains limited. Our study suggests that the microstructural development of the cerebral cortex in infants with CHD is abnormal in the newborn period compared with healthy controls and, importantly, that the degree of impairment is related to reduced CDO_2_. We speculate that hindered microstructural development underlies the abnormal macrostructural changes in brain development that have been observed through reduced birth head circumference,^49^ smaller brain volumes,^7^ and immature cortical folding^6^, ^10^, ^11^, ^12^, ^50^ and that strategies to optimize cerebral oxygenation in utero may offer the potential to ameliorate brain development in this population.

We found that cortical ODI was widely reduced in the CHD group, with associated but more sparsely distributed areas of higher FA. These findings suggest a hindered trajectory of normal brain development, with increased sensitivity to tissue changes using the more advanced NODDI model. As the brain matures in utero, cortical neurons migrate outward toward the pial surface, populating the cortex^51^ and resulting in a highly directional, parallel, columnar microstructure. This can be observed with diffusion tensor imaging as tensors with high FA, oriented radially to the cortical surface.^52^ As the cortex matures, an increasingly dense and complex cytoarchitecture forms, with dendritic arborization, glial proliferation, differentiation of radial glia, and synapse formation^51^, ^53^, ^54^ associated with an observed increase in cortical ODI.^39^ Increasing cytoarchitecture complexity also restricts water diffusion more evenly in all directions, with a consequent reduction in FA.^42^, ^52^ Cortical development between 25 and 38 weeks postmenstrual age shows a predominant increase in dendritic arborization and neurite growth (as represented by ODI), while between 38 and 47 weeks postmenstrual age it is dominated by increasing cellular and organelle density (as represented by NDI).^55^ This supports our finding that ODI was the most discriminating microstructural measure in this population, where developmental impairment of the brain is thought to originate in the third trimester.^4^, ^5^, ^6^, ^7^ The link between diffusion imaging studies and underlying tissue biology is supported by prior evidence that NODDI‐derived dispersion measures match their histological counterparts in adult postmortem specimens,^56^ and by correlations found between maturation of dendritic arbors at the cellular level and loss of diffusion anisotropy with cortical development in the rhesus macaque^16^ and fetal sheep.^57^

While reduced cortical folding complexity in newborns with CHD has previously been reported in our cohort^12^ and others,^11^, ^58^, ^59^ the link between cortical macrostructural and microstructural development in these infants has not been investigated previously. We found that the GI was positively associated with mean cortical ODI, independent of its association with increasing maturity. GI was also negatively but more weakly associated with mean cortical FA. Of interest, total brain volume was not associated with either FA or ODI. Taken together, these results suggest that macrostructural abnormalities observed in infants with CHD may be related to underlying impairments in dendritic arborization.

Changes in cortical orientation dispersion were more pronounced posteriorly than frontally, which is consistent with a previously described sequence of cortical development maturing earlier in the occipital cortex and completing later in frontal regions.^60^, ^61^, ^62^ Differences were also seen prominently in the region of the operculum, a region that has been repeatedly highlighted in infants with CHD. Findings of an “open operculum” with exposed insular cortex have been reported in CHD^50^, ^63^, ^64^, ^65^, ^66^ and have been associated with a poor outcome.^67^

Having established group differences between infants with CHD and healthy controls, we investigated the effect of CDO_2_ on development of cortical ODI. There was a widespread positive relationship between ODI and CDO_2_, supporting the hypothesis that impaired oxygen delivery to the developing brain may be associated with delayed cortical microstructural development. There was no relationship between ODI and either cerebral blood flow or preductal arterial saturation when considered individually, suggesting that both components of CDO_2_ are required to estimate oxygen delivery to the brain, and that when considered alone, neither component explains enough variance of ODI to achieve statistical significance. In the case of cerebral blood flow, this may additionally suggest that alternative proposed metabolic substrates^13^ may be less influential. These results support recent laboratory studies, with oxygen tension shown to regulate the development of human cortical radial glia cells. Moderate and severe levels of hypoxia exert negative effects on gliogenesis, mediated via reduced numbers of preoligodendrocytes and increased numbers of reactive astrocytes derived from cortical radial glia cells.^14^ Equally, diminished subventricular zone neurogenesis as a result of chronic hypoxia may represent a cellular mechanism that underlies immature cortical development in the CHD population.^15^ Taken together, these studies support the view that oxygenation of the developing brain is a crucial factor to optimize to restore the derailing trajectory of cortical development in this population. Future serial imaging to assess cortical development in CHD through infancy and early childhood would allow us to understand if microstructural differences observed in the newborn period are simply delayed with potential for catch‐up in later childhood, or if such abnormalities are permanent and persist into childhood. Such work may offer insights into whether early surgical repair may help prevent further divergence, resulting in better long‐term neurodevelopmental outcomes.

There were limitations to our study. First, quantitative estimates obtained from microstructural studies are invariably model dependent, exhibiting biases and limitations that are related to model assumptions. Despite this, NODDI indices have been shown to correlate with histological changes in neurite geometric configuration^56^ and offer a valuable proxy to underlying biological changes in microstructure. Second, CDO_2_ was also measured in the postnatal period, while the most influential period on brain growth would have been in utero, and particularly during the third trimester. Despite this, we feel that postnatal CDO_2_ remains a useful surrogate for severity of cardiac circulatory compromise to date, taking into account both measures of cerebral blood flow and degree of hypoxia as a result of structural changes in congenital heart disease. Third, the underlying genetic basis of CHD is becoming increasingly better understood^68^ and may represent a key contributor not only to structural heart disease and associated impaired CDO_2_ but also to intrinsic abnormalities in microstructural development of the brain. Future work in larger, more homogenous cohorts to characterize in utero flows, oxygen saturations, and CDO_2_ will allow further correlation of CDO_2_ or lesion type with measures of fetal brain development.

There are currently no validated neuroprotective therapies available for infants with CHD. Our demonstration that reduced CDO_2_ is associated with impaired cortical maturation in this population supports the development of strategies to optimize fetal CDO_2_. The provision of supplemental oxygen to mothers during pregnancy may enable restoration of fetal cerebral oxygen tension to levels required to prevent or reverse abnormal corticogenesis.^69^ This may be most valuable in cases of critical cyanotic CHD, where a severe reduction in CDO_2_ is most likely. In addition, the use of newer microstructural measures such as ODI may provide a crucial leading indicator in the postnatal period to assess the impact of novel interventions on cortical development before child neurodevelopmental outcomes can be assessed at a later age.

## Sources of Funding

This research was funded by the British Heart Foundation (FS/15/55/31649) and Medical Research Council UK (MR/L011530/1). This work received funding from the European Research Council under the European Union's Seventh Framework Programme (FP7/20072013)/ERC grant agreement no. 319456 (dHCP project), and was supported by the Wellcome Engineering and Physical Sciences Research Council Centre for Medical Engineering at Kings College London (WT 203148/Z/16/Z), MRC strategic grant MR/K006355/1, Medical Research Council Centre grant MR/N026063/1, and by the National Institute for Health Research Biomedical Research Centre based at Guy's and St Thomas’ NHS Foundation Trust and Kings College London. Dr O'Muircheartaigh is supported by a Sir Henry Dale Fellowship jointly funded by the Wellcome Trust and the Royal Society (206675/Z/17/Z). The views expressed are those of the authors and not necessarily those of the NHS, the National Institute for Health Research, or the Department of Health.

## Disclosures

None.

## Supporting information


**Table S1.** Differences in Mean Cortical Microstructure and Cerebral Oxygen Delivery Between Those With and Without Punctate White Matter Lesions
**Table S2.** Differences in Mean Brain Volume, Regional Brain Volumes, and Mean Diffusion Measures, Between Those With Congenital Heart Disease (CHD) and Age‐Matched Controls
**Figure S1. A**, Mean orientation dispersion index (ODI) and (**B**) fractional anisotropy (FA) from significant cortical regions plotted against gestational age at scan, for both congenital heart disease (CHD; open blue marker, n=48) and control (closed orange marker, n=48) groups.
**Figure S2.** Infants with congenital heart disease (CHD, n=37) exhibit impaired orientation dispersion index compared with healthy age‐matched controls (n=37), overlaid on the mean orientation dispersion index (ODI) template.
