# Supplementary material for: Abnormal Microstructural Development of the Cerebral Cortex in Neonates With Congenital Heart Disease Is Associated With Impaired Cerebral Oxygen Delivery
Source: J Am Heart Assoc. 2019 Mar 1;8(5):e009893. doi: 10.1161/JAHA.118.009893 (PMC6474935; doi:10.1161/JAHA.118.009893)
Supplement: Supplementary file 1 — Table S1. Differences in Mean Cortical Microstructure and Cerebral Oxygen Delivery Between Those With and Without Punctate White Matter Lesions Table S2. Differences in Mean Brain Volume, Regional Brain Volumes, and Mean Diffusion Measures, Between Those With Congenital Heart Disease (CHD) and Age‐Matched Controls Figure S1. A, Mean orientation dispersion index (ODI) and (B) fractional anisotropy (FA) from significant cortical regions plotted against gestational age at scan, for both congenital heart disease (CHD; open blue marker, n=48) and control (closed orange marker, n=48) groups. Figure S2. Infants with congenital heart disease (CHD, n=37) exhibit impaired orientation dispersion index compared with healthy age‐matched controls (n=37), overlaid on the mean orientation dispersion index (ODI) template. [file JAH3-8-e009893-s001.pdf]

SUPPLEMENTAL MATERIAL

Table S1. Differences in mean cortical microstructure and cerebral oxygen delivery between those with and without punctate white matter lesions.

	No punctates (n=33)	Punctate lesions (n=15)	P-value
**Mean cortical FA**	0.12	0.12	0.342
**Mean cortical MD ($10^{-3}mm^2.s^{-1}$)**	1.16	1.18	0.355
**Mean cortical NDI**	0.27	0.26	0.317
**Mean cortical ODI**	0.48	0.47	0.062
**Cerebral oxygen delivery (ml/min)**	1876	1716	0.654

Groups compared using a general linear model using post-menstrual age as a covariate. FA indicates fractional anisotropy; MD, mean diffusivity; NDI, neurite density index; ODI, orientation dispersion index.

Table S2. Differences in mean brain volume, regional brain volumes, and mean diffusion measures, between those with congenital heart disease (CHD) and age-matched controls.

	CHD (n=48)	Controls (n=48)	P-value
**Total brain volume (ml)**	311.3	330.0	0.001
**Cortical grey matter volume (ml)**	122.3	130.5	0.001
**Frontal grey matter volume (ml)**	41.3	43.4	0.008
**Parietal grey matter volume (ml)**	28.4	30.5	< 0.001
**Temporal grey matter volume (ml)**	25.6	27.5	< 0.001
**Occipital grey matter volume (ml)**	18.7	20.2	0.002
**Whole cortex mean FA**	0.1171	0.1160	0.311
**Whole cortex mean MD ($10^{-3}mm^2.s^{-1}$)**	0.0012	0.0012	0.212
**Whole cortex mean NDI**	0.2652	0.2666	0.496
**Whole cortex mean ODI**	0.4740	0.4840	0.002
**Significant regions only mean FA**	0.1266	0.1140	n/a
**Significant regions only mean ODI**	0.4605	0.4897	n/a

Groups compared using a general linear model using post-menstrual age as a covariate. Mean values of FA/ODI for regions of difference identified during voxel-wise analysis are presented for descriptive purposes. FA indicates fractional anisotropy; MD, mean diffusivity; NDI, neurite density index; ODI, orientation dispersion index.

Figure S1. (a) Mean orientation dispersion index (ODI) and (b) fractional anisotropy (FA) from significant cortical regions plotted against gestational age at scan, for both congenital heart disease (CHD, open blue marker, n=48) and control (closed orange marker, n=48) groups.

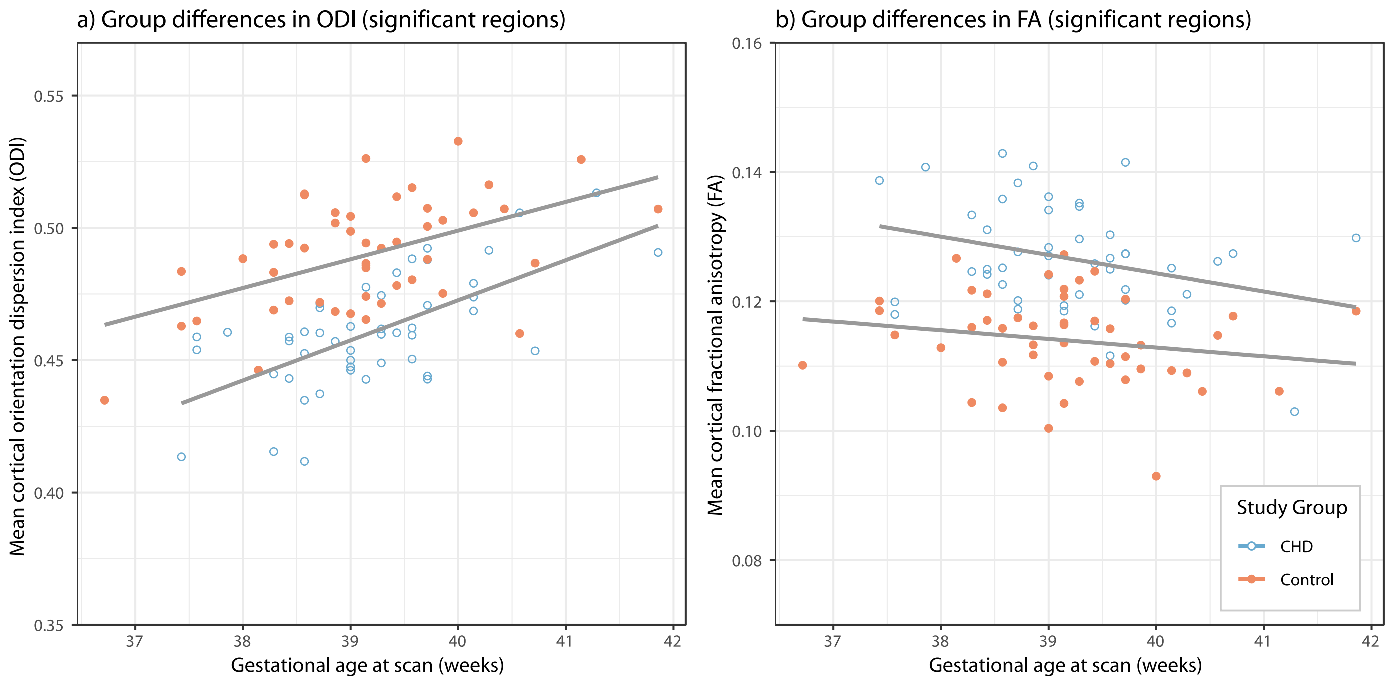

Significant cortical regions identified by cross-subject voxel-wise statistical analysis performed using FSL Randomise v2.9, with a general linear model (GLM) used to assess group differences (as demonstrated in Figure 1).

Figure S2. Infants with congenital heart disease (CHD, n=37) exhibit impaired orientation dispersion index compared to healthy age-matched controls (n=37), overlaid on the mean orientation dispersion index (ODI) template.

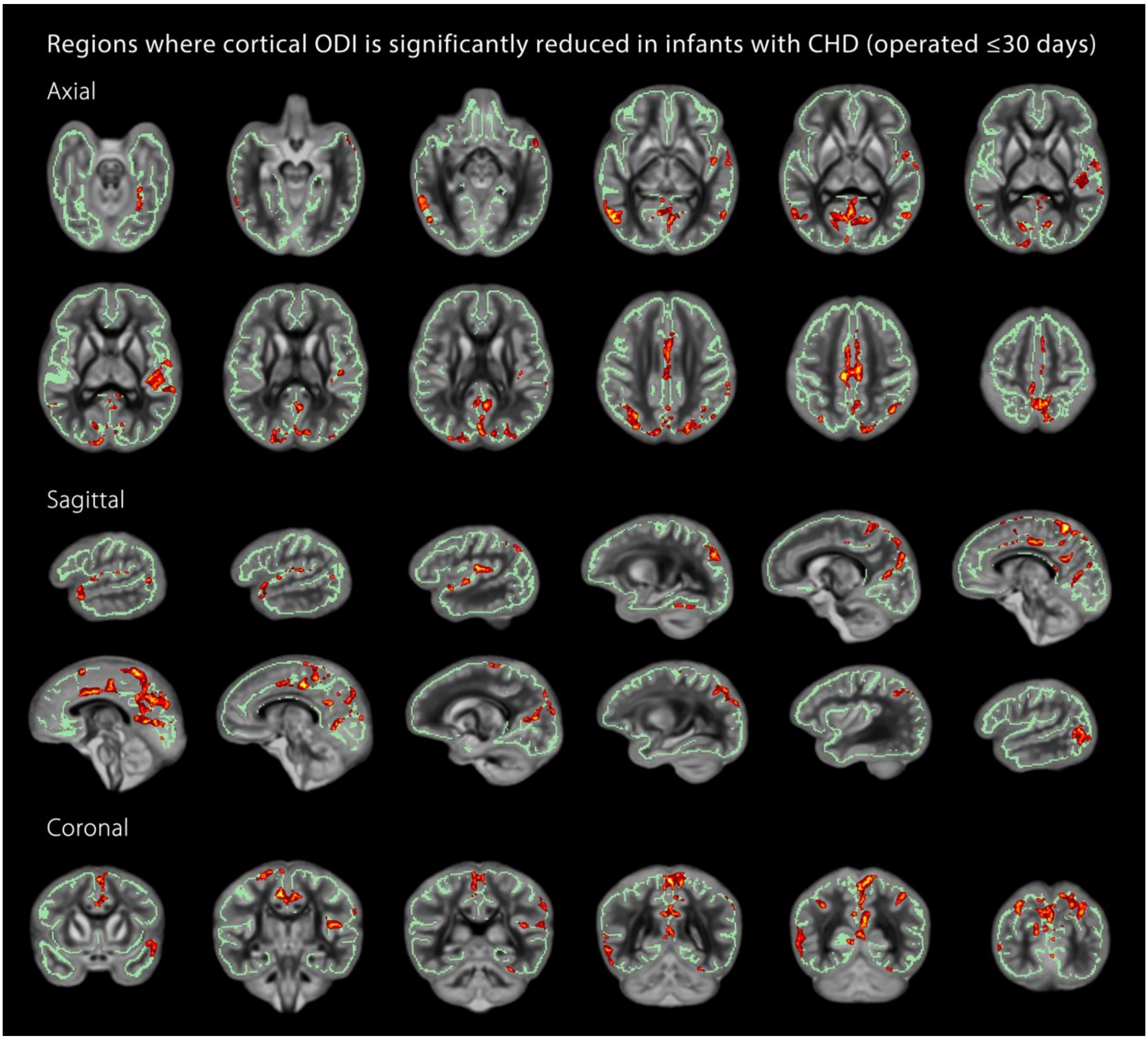

Red-Yellow indicates P < 0.05 after family-wise error correction for multiple comparisons following threshold-free cluster enhancement. Results are shown overlaid on the mean cortical skeleton (green). Cross-subject voxel-wise statistical analysis performed using FSL Randomise v2.9, with a general linear model (GLM) used to assess group differences between diffusion measures of infants with CHD and healthy controls. Both analyses included gestational age at birth and at scan as covariates. Number of permutations was 10,000. Left–right orientation is according to radiological convention.
